# 2-Ethylhexyl Diphenyl Phosphate (EHDPP) Induces Hepatic Expression of Cytochrome P450s, Liver Damage, and Genotoxicity in Mice

**DOI:** 10.3390/toxics14080691

**Published:** 2026-08-05

**Authors:** Zhao Zhou, Hongbin Gao, Shunda Zhu, Lvlue Cai, Yijing Chen, Keqi Hu, Yungang Liu

**Affiliations:** 1 Guangdong Provincial Key Laboratory of Tropical Disease Research, Department of Toxicology, School of Public Health, Southern Medical University, 1023 S. Shatai Road, Guangzhou 510515, China; zhouzhao2022@163.com (Z.Z.); z22420129@i.smu.edu.cn (S.Z.); chenyijing@gwcmc.org (Y.C.); 2 Guangdong Provincial Biotechnology Research Institute, 11 Fengxin Road, Science City, Guangzhou 510663, China; ghb@gdlami.com (H.G.); cnl1351731069@163.com (L.C.); 3 Department of Science and Education, Guangdong Second Provincial General Hospital, 466 Xingang Middle Road, Guangzhou 510317, China

**Keywords:** comet assay, cytochrome P450 (Cyp) enzymes, 2-ethylhexyl diphenyl phosphate (EHDPP), flame retardant, hepatotoxicity, micronucleus test

## Abstract

As a commonly present organophosphorus flame retardant and persistent organic pollutant, 2-ethylhexyl diphenyl phosphate (EHDPP) has been observed to be genotoxic in cultured human hepatoma (HepG2) cells which depends on CYP activities. Yet, its impacts on hepatic Cyp expression, hepatotoxicity and genotoxicity in intact mammalians remain unidentified. In this study, adult male C57BL/6J mice received EHDPP by gastric gavage at doses of 50, 100, and 150 mg/kg (b.w.)/d for 7 d, then the hepatic expression of several Cyp proteins, aryl hydrocarbon receptor (AhR) and pregnane X receptor (PXR) was analyzed by Western blotting; hepatoxicity was determined by serum ALT/AST activities and hepatic histological examination, while genotoxicity by comet assay, phosphorylated histone (γ-H2AX) protein, micronucleus test, and Pig-a assay. A micronucleus test in mouse hepatoma (Hepa1-6) cells in vitro was employed to observe the modulating effect of PCB 126 (100 nM)/BAY-218 (700 nM) (Ahr-Cyp1a1 activator/inhibitor). The results indicated that EHDPP induced hepatic Cyp1a1, 2e1, AhR, Cyp1a2, Cyp3a4 and PXR proteins and histologic liver damage at 50 mg/kg/d and/or higher doses, while at the highest dose (150 mg/kg/d) with hepatic DNA damage and micronucleus formation in bone marrow polychromatic erythrocytes. The result of Pig-a assay (at 14 and 28 d) was negative. In Hepa1-6 cells EHDPP induced micronucleus marginally; however, this effect was enhanced by PCB 126, while abolished by BAY-218. This study suggests that EHDPP may enhance protein expression of hepatic Cyp1a1, Cyp2e1, AhR and PXR and induce liver damage and DNA/chromosome damage in mice; Cyp1a1 might be a major activating enzyme.

## 1. Introduction

2-Ethylhexyl diphenyl phosphate (EHDPP) is a commonly consumed organophosphorus flame retardant (OPFR). Owing to its highly preferred endurance, flexibility, and heat resistance, EHDPP has been largely used in the production of textiles, homewares, paints, and plastic products [1,2]. Particularly, along with the inclusion of polybrominated diphenyl ethers (PBDEs, previously dominating the flame retardant markets) on the list of persistent organic pollutants as documented in the Stockholm Convention, PBDEs have been gradually phased out, alternative to which EHDPP has become one of the new generation flame retardants. EHDPP and other OPFRs are commonly present in various environmental media, such as the surface water, indoor dusts, sedimentations, and biota [3,4], and they have been detected in specimens from human populations (breast milk, blood, and urine) and other organisms (such as marine life and birds) [5,6]. EHDPP has been approved for use in the production of food-packaging materials, and it may enter the human body through ingestion of contaminated food, inhalation of dusts containing EHDPP, and skin contact with relevant materials, among which dietary intake is supposed to be the major mode of exposure. Due to human activities and long-distance transmission through air flow, EHDPP is distributed in the world very widely. For example, in the soil and sediments in Tibet, China, EHDPP is dominant in various OPFRs [7]. Notably, EHDPP concentrations in processed food appeared to be significantly higher than in unprocessed food, which implies that EHDPP may contaminate food through the use of packaging materials [8]. For a population in Beijing, China, the median concentrations of EHDPP in whole blood and serum were 1.10 and 0.93 ng/mL, respectively [9]. Meanwhile, epidemiological and toxicological investigations suggest that EHDPP may have potential toxicity in humans and/or other organisms, which includes reproductive [10], developmental [11], and hepatic toxicity [1,12], as well as participating in chemical carcinogenesis [1]. Although there has been no human population-based cohort study verifying the carcinogenicity of EHDPP, cross-sectional investigations suggest that the blood burden of EHDPP (and some other OPFRs) is significantly associated with several cancers (such as lung cancer and some female-specific cancers) [13,14]; yet the mutagenicity of EHDPP in mammals, particularly under in vivo exposures, remains unclear.

Gene mutation and chromosome damage (major endpoints of mutagenesis) are contributive to chemical carcinogenesis, while DNA damage (in various forms, such as DNA breakage, formation of DNA adducts, base alkylation, and DNA cross-linking) is the prerequisite for gene mutations and structural chromosome alterations [15,16], as well as some tissue damage [17]. EHDPP by itself is incapable of forming covalent bonds with DNA and proteins, while only after metabolic activation (mostly requiring catalysis by relevant xenobiotic-metabolizing enzymes, such as cytochrome P450s (CYPs)) can it be converted to bioreactive metabolites. Regarding the metabolism of EHDPP, in an early study the metabolic fate of ^14^C-EHDPP orally administered to male rats was investigated. The results indicated rapid absorption, and within 24 h most radioactivity was excreted in the urine and feces, all excreted by 7 d; the radioactivity was widely distributed, relatively high in the blood, liver, kidneys and adipose tissue. The major metabolites in the urine were diphenyl phosphate (DPHP) and phenol, with *p*-hydroxyphenyl DPHP (OH-DPHP) and monophenyl phosphate (MPP) as minor metabolites [18]. In a population of east China, the blood levels of EHDPP and DPHP were dominant among various organophosphorus triester and diester compounds, respectively [19]. Further multivariant linear regression analysis of the data indicated that the concentrations of EHDPP and DPHP were significantly correlated with each other, supporting that DPHP is a major metabolite of EHDPP; meanwhile, from the blood samples other metabolites (presumably formed from EHDPP) were also detected, such as 2-ethylhexy monophenyl phosphate (EHMPP) and hydroxylated EHMPP (OH-EHMPP) [19]. These hydroxylated metabolites may not be bioreactive enough to attack DNA and proteins by forming covalent bonds, and whether they are further metabolically activated into electrophilic metabolites remains unidentified, especially considering that, the more reactive an electrophile is, the shorter lived and more difficult to be detected it would be. Indeed, we have previously observed that EHDPP induced DNA- and chromosome-breaking effects in cultured mammalian cells, which depend on metabolic activation by several human CYP enzymes, predominantly CYP2E1 and CYP3A4 [20]. However, relevant genotoxic effects in intact mammalians have never been reported.

In this study, the hepatoxicity and genotoxicity of EHDPP, and the concomitant modulation of the hepatic expression of some nuclear receptors and Cyp enzymes, were determined with a subacute exposure model in mice. Moreover, an in vitro micronucleus test with EHDPP as the test compound was performed, with and without modulators of a speculatively involved Cyp, to explore the activating enzyme as required for the genotoxicity of EHDPP.

## 2. Materials and Methods

### 2.1. Chemicals and Materials

EHDPP (purity ≥ 97%) and *N*-nitrosodiethylamine (NDEA, purity ≥ 99%) were purchased from Shanghai Aladdin Biochem. Technol. Co., Ltd. (Shanghai, China). 3,3′,4′,4′,5-Pentachlorobiphenyl (PCB 126, ≥99%) was from AccuStandard Inc. (New Haven, CT, USA). (S)-6-(4-Chlorophenyl)-2-(3-fluorophenyl)-N-(1-hydroxypropan-2-yl)-3-oxo-2 (BAY-218, ≥98%) was from Meilunbio (Dalian, China).

The primary antibody raised in rabbits against human CYP3A4 (cross-reacting with mouse Cyp3a11) and the secondary antibody, i.e., horseradish peroxidase (HRP)-conjugated goat anti-rabbit IgG, were both purchased from Abcam (Cambridge, UK). The primary antibodies raised in rabbits against human aryl hydrocarbon receptor (AhR), human pregnane X receptor (PXR), human CYP1A1 (cross-reacting with mouse Cyp1a1), human CYP1A2 (cross-reacting with mouse Cyp1a2), and human phosphorylated histone 2AX (γ-H2AX), which cross-react with mouse AhR, PXR, Cyp1a1, Cyp1a2, and γ-H2AX, respectively, were from ABclonal (Wuhan, China). The primary antibody raised in rabbits against human CYP2E1 (cross-reacting with mouse Cyp2e1) was from Proteintech Group, Inc. (Wuhan, China). The primary antibody raised in rabbits against human GAPDH (cross-reacting with mouse GAPDH) was from Yeasen Biotechnology (Shanghai, China). APC-labeled anti-mouse CD24 antibody (APC anti-mouse CD24) was from Biolegend (San Diego, CA, USA), and SYTO™13 (for staining nucleic acid) was from Thermo Fisher Scientific (Carlsbad, CA, USA).

The assay kits for serum alanine aminotransferase (ALT) and aspartate aminotransferase (AST) activities were purchased from Elabscience Biotechnology Co., Ltd. (Wuhan, China), and the Comet Assay Kit was from Beyotime (Shanghai, China). The Cell Counting Kit-8 (CCK-8) was obtained from Selleck Chemicals (Houston, TX, USA). In the in vitro experiments, each test compound was dissolved in dimethyl sulfoxide (DMSO) before exposing cell cultures, with the final concentration of DMSO being kept at 0.1% (*v*/*v*).

### 2.2. Animals and Treatments

Five-week-old male C57BL/6J mice (totally 40 mice, not including those used in the pre-experiment) were purchased from Guangdong Medical Laboratory Animal Center (Guangzhou, China, License No. SCXK (Yue) 2021-0002), then they were acclimatized to the animal (specific-pathogen-free) laboratory in Southern Medical University for 1 week, during which they had access to standard feeds and tap water ad libitum. The animal room was maintained at 20–26 °C and 55–65% humidity with a 12 h light/12 h dark cycle. All manipulations of the animals were in accordance with the National Institutes of Health guidelines outlined in the Guide for the Care and Use of Laboratory Animals and were approved by the Experimental Animal Ethics Review Committee of Southern Medical University (Code: SMUL202408036). Female mice were not used for fear of potential impact from periodic endocrine fluctuations.

The design of the chemical exposure regimen was in accordance with the purpose of exploration of the threshold level of EHDPP for genotoxicity, a major mechanism of chemical carcinogenesis, in mammalians under standard exposure durations for the endpoints of several genotoxicity tests.

**Figure 1:**
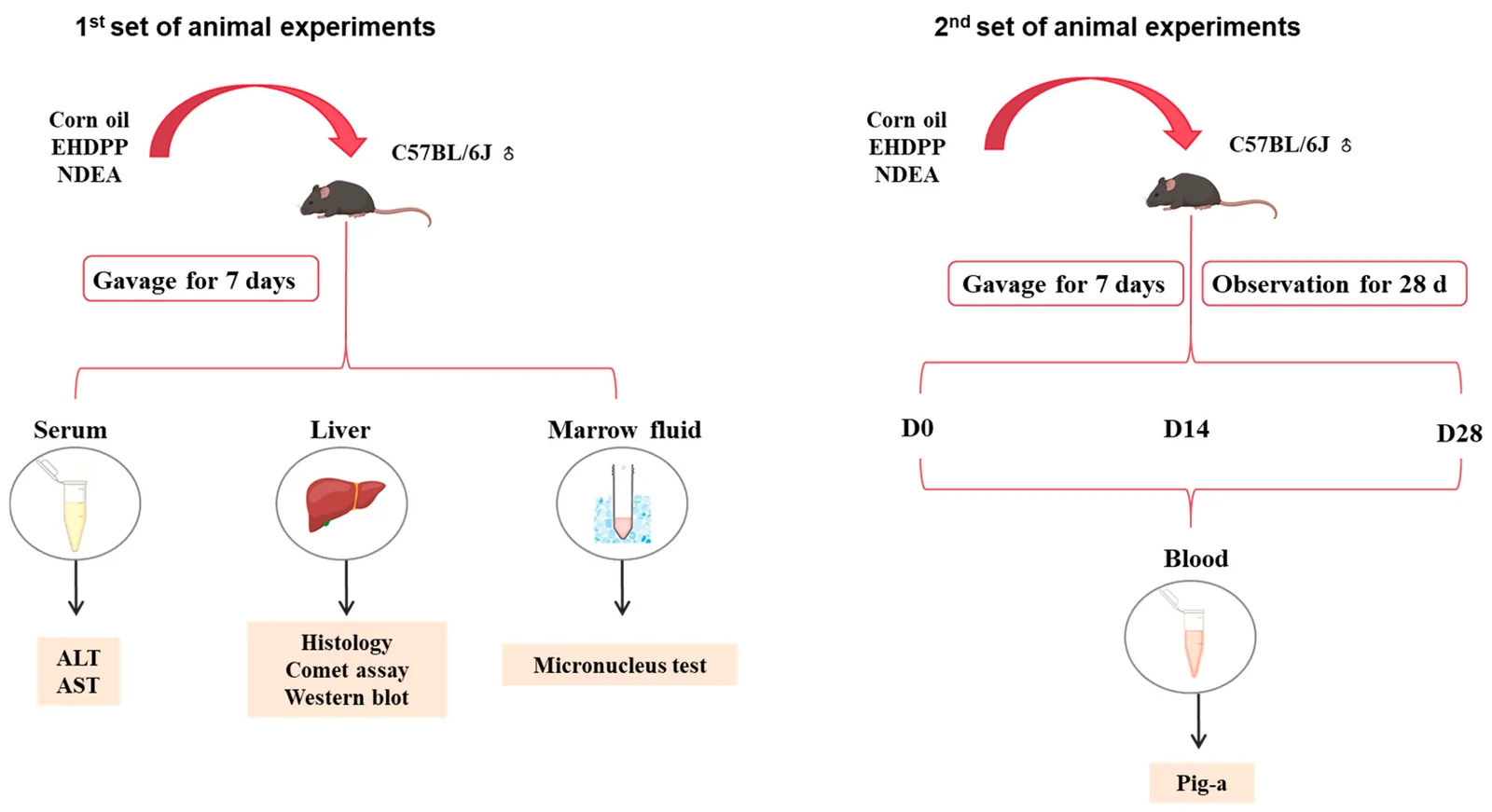
A schematic diagram of the animal experimental design.

In the first set of experiments, a 7 d exposure was chosen since it is standard for the Mammalian Erythrocyte Pig-a Gene Mutation Assay established by the Organization for Economic Co-operation and Development (OECD) [21], while it was slightly extended from the typical 3 d exposure for the test of the other genotoxicity endpoints. Six-week-old (young adult) male C57BL/6J mice were randomly divided into 5 groups, with 5 mice in each group, which included the control (only corn oil as a solvent), EHDPP low dose (50 mg/kg b.w./d), EHDPP middle dose (100 mg/kg/d), EHDPP high dose (150 mg/kg/d), and positive control (NDEA, 15 mg/kg/d) group (so 25 mice in total were used). The treatments lasted for seven consecutive days, during which the administration of test compounds was performed by gastric gavage at 8:00~9:00 in the morning. On day 8, all mice were subjected to sacrifice, hepatic histology/serum aminotransferase assay, determination of hepatic levels of various proteins, and a bone marrow micronucleus test, which are described in the following sections. A schematic diagram is shown in Figure 1 (left panel).

A second set of experiments followed the same treatment regimen as described above, while with only 3 mice in each treatment (so, totally, 15 mice were involved). A Pig-a test was performed on days 0, 14, and 28 by using the blood collected from the orbital venous plexus of each animal (see Figure 1, right panel).

During the whole experimental process, each mouse was observed for general condition and activity daily, and its body weight was measured every 3 days.

### 2.3. Animal Necropsies and Sample Collection

For the first set of experiments, on day 8 each mouse was euthanized by a dose of 40 mg/kg of pentobarbital *via* intraperitoneal injection. Then about 600~700 μL of fresh blood was collected from each mouse by a puncture into the abdominal aorta, for subsequent serum isolation and ALT/AST activity measurement. Then each mouse was killed by cervical dislocation.

From each mouse, the liver was freshly isolated and weighed (dividing the liver wet weight by the body weight produced the liver organ coefficient), then an approximately 5 mm thick strip of hepatic tissue was cut off and fixed in 4% paraformaldehyde for histopathological analysis. Another block (about 5 mm^3^ in size) of hepatic tissue was excised from the left lateral lobe of the liver for the preparation of hepatocytes and comet assay. An aliquot of about 15 mg liver tissue was made from the liver of each mouse, which was immediately frozen in liquid nitrogen, for subsequent preparation of protein lysate and Western blot assays of various hepatic proteins. Meanwhile, the femurs of each mouse were isolated for subsequent marrow fluid collection and micronucleus test (to be described in Section 2.9).

For the second set of experiments, on days 0 (immediately after treatment), 14 and 28 after the treatment of mice with test compounds, each mouse was anesthetized by intraperitoneal injection of 0.3% sodium phenobarbital at a dose of 40 mg/kg, then 100~200 µL blood was collected from each mouse by a puncture into the orbital venous plexus, followed by cotton ball compression for hemostasis. After the final sampling of blood on day 28, each mouse was killed by cervical dislocation. The fresh blood was anticoagulated by regular treatment with K_2_-EDTA and subsequently used in a Pig-a assay.

### 2.4. Serum ALT/AST Activity Measurement

Serum was prepared using the fresh blood sample collected from each mouse through natural clotting (at room temperature), subsequent centrifugation at 12,000× *g* for 15 min (4 °C), and harvest of the supernatant. The serum activity of ALT or AST was measured in accordance with the assay kit manufacturer’s instructions. Briefly, after adding sample diluent (40 µL) and serum sample (10 µL) sequentially into each well of a 96-well cell culture board and mixing them by gentle shaking, 100 µL of enzyme-labeled reagent was further added, followed by closing the wells with Parafilm and incubation at 37 °C for 1 h. Then, to each well, 300 µL of washing liquid was added, and after standing for 1 min, the liquid mixture was removed. This washing procedure was repeated 5 times. Afterwards, into each well, color developers A and B (50 µL for each) were sequentially added, followed by gentle shaking to mix them, then the culture board was placed in the dark at 37 °C for 15 min to permit color development. The reaction was stopped by the addition of 50 µL stop solution to each well, and optical density was measured immediately at 515 nm by a microplate reader (Tecan, Spark 20M, Männedorf, Switzerland). Triplicate measurements were set up for each sample, and serum ALT/AST activity (U/L) was calculated in accordance with the paralleling standard curve.

### 2.5. Histopathological Examination of the Liver Tissue

Following fixing each hepatic sample in 4% paraformaldehyde, further treatment of each sample, section preparation, a series of treatments of the sections, and staining of the sections with hematoxylin and eosin (H&E) were performed in accordance with our recent report [22]. The stained sections mounted on slides were histologically read under the double blind principle by an experienced experimenter using a light microscope (Olympus, CX31, Tokyo, Japan). Histopathological changes in the liver were classified into grades 0, 1, 2, and 3 according to established criteria, which mark escalating acute hepatic response such as hepatocyte enlargement and disarrangement, acidophil body formation, and infiltration of various inflammatory cells, respectively [23], based on evaluation of 10 fields randomly selected from the slide/slides of each sample. Data are expressed as the distribution of various histopathological grades in each group.

### 2.6. Western Blot Analyses of AhR, PXR, Cyp1a1, Cyp1a2, Cyp2e1, Cyp3a11, and γ-H2AX

An aliquot of liver tissue from each mouse was taken out from liquid nitrogen tank, and after being defrosted at room temperature, it was cut with ophthalmic scissors into very small pieces, followed by homogenization of each sample, preparation of protein lysate, protein quantification of the lysate, and Western blot assays of various hepatic proteins, according to our recent reports [22]. Particularly, an amount of protein lysate containing 30 μg protein was loaded onto each lane of 10% SDS-PAGE, as appropriate according to pre-experiment results; after the proteins were separated by electrophoresis, they were blotted onto a polyvinylidene fluoride (PVDF) membrane, which was blocked in tris-buffered saline with 0.1% Tween 20 (TBST) containing 5% non-fat milk for 1 h, and then incubated with various primary antibodies, including those raised in rabbits against human AhR (1:2000 diluted), human PXR (1:3000), human CYP1A1 (1:2000), human CYP1A2 (1;1000), human CYP2E1 (1:5000), human CYP3A4 (1:5000), human γ-H2AX (1:2000), and human GAPDH (1:20,000) at 4 °C overnight. Each protein was probed with horseradish peroxidase (HRP)-conjugated secondary antibodies for 1 h at room temperature, then they were visualized using an enhanced chemiluminescence (ECL) detection system. Each target protein was quantified with the ImageJ software (version 1.52a) relative to that in the control group, where GAPDH was used as the loading control. Two or three replicates were set up in each experiment, which was independently repeated 3 times, in order to verify the data consistency.

### 2.7. Comet Assay

Each freshly isolated block of hepatic tissue sample was soaked in an appropriate volume of ice-cold PBS solution, then it was cut into very small pieces. Separated hepatocytes were prepared, then subjected to a comet assay (alkaline single-cell gel electrophoresis) according to our recent descriptions [22]. In each experiment three independent replicates were set up. The resultant slides were blindly coded and, using a fluorescence microscope (Olympus, model BX53, Tokyo, Japan), the images on each slide were captured, where 10 different fields under a 20× object lens (comprising at least 100 cells) per slide were randomly selected for measurement of fluorescence signals. A Comet Assay Software Project (Beijing Biolaunching Technologies Co., Ltd., Beijing, China) was used to calculate the Tail Moment and Tail DNA (%).

### 2.8. Pig-a Mutagenicity Assay

Pig-a mutagenicity assay was performed according to established conditions [24], with minor modifications. Briefly, an aliquot of 80 µL freshly prepared anticoagulated blood was taken, to which 100 µL of 1% heparin was added. The mixture was gently added to 3 mL of lymphocyte separation medium, which was centrifuged at 1500 RPM for 20 min at room temperature (25 °C). The pellet (cells) was resuspended in 150 µL of cold (4 °C) PBS. The cells were spun down by centrifugation at 240× *g*, then resuspended in 5 mL of cold PBS supplemented with 2% fetal bovine serum (FBS). This cell washing procedure was repeated one more time, then the cells were resuspended in 150 µL of cold PBS (with no FBS), to which appropriate amount of anti-CD24-APC working solution (anti-CD24-APC: PBS as 1:30, *v*:*v*) was added; after being mixed, the reaction mixture was placed on ice in the dark for 30 min (staining). Subsequently, the mixture was centrifuged at 1500 RPM for 5 min at room temperature, followed by cell washing treatment twice (as described above), then the cells were resuspended in 1 mL of SYTOTM13 working solution (final concentration being 157 nM) and subjected to staining in darkness for 30 min. Finally, the sample was kept in ice and was analyzed with 10^6^ cells per sample by flow cytometry (BD LSRFortessa X-20, San Jose, CA, USA) within 2 h. Cells stained for CD24 were regarded as glycosylphosphatidylinositol (GPI) positive (wild-type), while those negative for the staining were recognized as GPI negative (mutants) which reflects a phenotypic loss of GPI-anchored proteins, commonly resulting from mutations in the Pig-a gene. Data are expressed as the frequency of mutants in each treatment relative to that in the control.

### 2.9. In Vivo Micronucleus Assay

The femurs from each mouse were cut on both upper and lower ends, then the bone marrow was perfused with an appropriate amount of Dulbecco’s modification of Eagle’s medium (DMEM, with 4.5 g/L glucose) (Gibco, Carlsbad, CA, USA) supplemented with 10% (*v*:*v*) fetal bovine serum (FBS) (Gibco, Biological Industries, Beit HaEmek, Israel) for collection of bone marrow. The bone marrow was then smeared on glass slides, fixed in methanol/acetic acid (3:1), and stained with Giemsa stain (at pH 6.8), as previously described [25]. The slides were blindly scored by an experienced experimenter under light microscopy at 1000× magnification: (1) totally 2000 randomly encountered polychromatic erythrocytes (PCEs) were observed for the presence or absence of micronucleus, from which the frequency of micronucleated PCE (‰) was calculated by dividing the number of micronucleated PCEs by the total number of PCEs observed (2000); (2) a total of 1000 randomly encountered erythrocytes were classified as either PCEs or normal chromatic erythrocytes (NCEs), from which the percentage of PCEs in the total number of both PCEs and NCEs was calculated, indicating the level of cell proliferation in the bone marrow (propagability of hematopoietic cells is essential for the validity of a bone marrow micronucleus test).

### 2.10. Cell Line, Cell Culture, and Cytotoxicity Test

Hepa1-6 is a cell line established from mouse hepatocellular carcinoma, with a cell population doubling time of about 24 h. The cells were cultured in DMEM supplemented with 10% FBS, 100 IU/mL penicillin G and 100 μg/mL streptomycin at 37 °C in a humidified atmosphere containing 5% CO_2_.

The level of cell viability and growth was determined using the CCK-8 assay, in which the intracellular content of NADH was measured according to the optical density at 450 nm. The test was performed following previous descriptions [26], where the values from CCK-8 assay for growing cells were well correlated with the elevating cell counts (evidencing the representativeness of cell proliferation and viability by CCK-8 assay), with modifications only to the chemical exposure regimen. Briefly, the cells were exposed to EHDPP at concentrations ranging from 5 to 40 μM for 48 h, with DMSO (0.1%, *v*:*v*) being used as the solvent. As modulators of specific Cyp enzymes, PCB 126 (100 nM, activator of AhR) and BAY-218 (700 nM, antagonist of AhR) were added to some cultures from 6 h after cell inoculation (18 h ahead of EHDPP exposure) to the end of the regimen (totally for 66 h). Six independent replicates were set up in each treatment. Finally, 10 μL of CCK-8 was added to each culture, which was maintained at 37 °C for 2 h, then optical density was measured at 450 nm by a microplate reader (Biorad Model 680, Hercules, CA, USA).

### 2.11. In Vitro Micronucleus Test

The micronucleus test in Hepa1-6 cells was performed in accordance with previous descriptions [27], with a chemical exposure regimen adapted to that described in Section 2.10. EMS (2.5 mM, a directly acting genotoxicant) was used as the positive control. Duplicate cultures were set up in each group. The resultant slides were blindly scored by an experienced experimenter under microscopy at 1000× magnification. In each experiment a total of 2000 randomly encountered and structurally integrated interphase cells were observed for the presence of micronucleus in the cytoplasm, thus the frequency of micronucleated cells (‰) was obtained. Data are expressed as means ± half ranges of variation in each group.

### 2.12. Statistical Analysis

The serum aminotransferase activities, Tail Moment and Tail DNA%, frequency of micronucleated PCEs, relative level of each hepatic protein, and cell viability/growth of cultured cells are expressed as means ± S.D.; after each set of data was tested for the normality of distribution, they were statistically analyzed by using the one-way ANOVA; in the case of statistical significance (*p* < 0.05), the data were further subjected to Dunnett’s test, with the group with only the solvent being used as the control. The duplicate data from the in vitro micronucleus test, however, were combined to form quantal data as valid for a χ^2^ examination. The distribution of hepatic histopathological grades in each treatment was analyzed by a rank sum test.

## 3. Results

### 3.1. Effects of EHDPP on the Body Weight, Liver Organ Coefficient, Serum Aminotransferase Activities and Hepatic Histology in Young Adult Male C57/BL6J Mice

**Figure 2:**
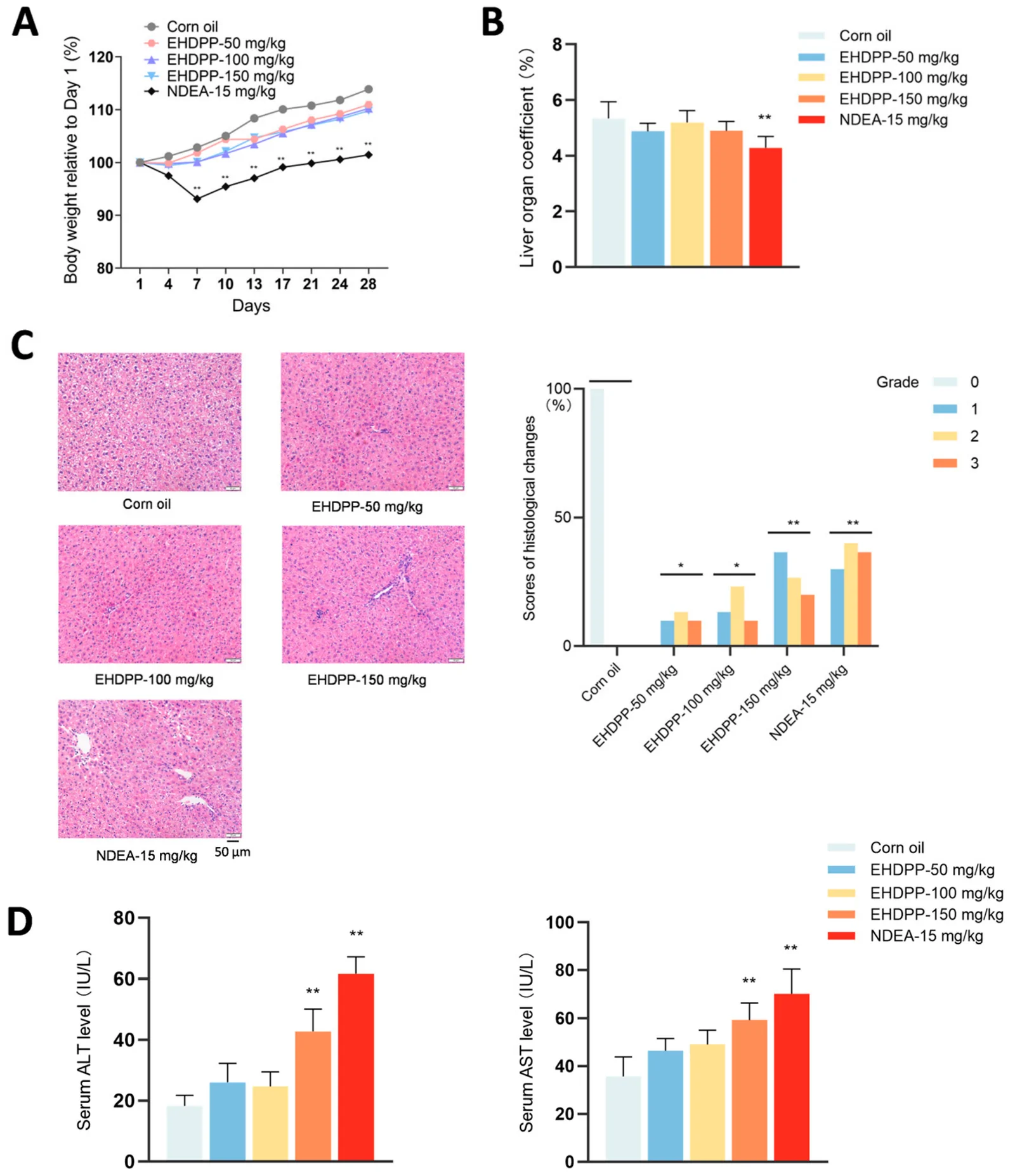
Effect of EHDPP on the body weight, liver organ coefficient, activities of serum aminotransferases, and hepatic histologic structure. Six-week-old C57BL/6J mice were exposed to EHDPP by gastric gavage at doses of 50, 100, and 150 mg/kg/d for 7 days, where corn oil was used as the vehicle, and NDEA (15 mg/kg/d, an indirect genotoxicant requiring metabolic activation primarily by Cyp2e1) served as a positive control, with five mice in each group. The data of body weights (panel (**A**)) were based on the second set of animal experiments (n = 3) which lasted for 28 d (the values on days 1, 4, and 7 were similar to those in the first set of animal experiments, which are not presented in the figure), while the other data, including liver organ coefficient (**B**), hepatic histological changes (under microscopy at 200× magnification) (**C**), and serum aminotransferase activities (**D**), were all from the first set of experiments as observed at 8 d. Data were statistically analyzed using either a one-way ANOVA (**A**,**B**,**D**) or a rank sum test (**C**); * *p* < 0.05, ** *p* < 0.01, as compared with the control.

During the whole experimental duration, the body weights of mice in the control group increased gradually, and there was a similar tendency in the groups with EHDPP at various doses (no statistically significant difference at any time points from the solvent control was observed); however, the body weights of mice in the positive control were decreased with statistical significance from 7~28 d as compared with the solvent control (Figure 2A). Meanwhile, using a variance trend test to compare the trend of body weight change during the whole 28-day experiment between different groups, statistically significant differences were present between the (solvent) control and positive control (*p* = 0.005), and between 50 mg/kg/d EHDPP and the positive control (*p* = 0.22), but not in any other comparisons (not shown in Figure 2). The results suggest a toxic effect of positive control (NDEA) on the experimental animals, while EHDPP at various doses did not show significant influence on the body weight. Likewise, the liver organ coefficients in mice exposed to EHDPP at various doses were not significantly different from that in the control (*p* > 0.05), nevertheless, that in the positive control was significantly lower than in the (solvent) control, again indicative of some adverse effect on the liver (Figure 2B). As shown in Figure 2C, the hepatic histology in the solvent control was 100% in grade 0, while in the mice exposed to increasing doses of EHDPP, and 15 mg/kg/d of NDEA, there appeared increasing frequencies of grade 1, 2, and 3 histologic changes, with significant difference with the control group, which indicates an histologic effect of EHDPP on the hepatic microstructure. Finally, as shown in Figure 2D, the serum ALT and AST activities (marker of hepatocyte damage) in mice exposed to 50 and 100 mg/kg/d of EHDPP were not different from those in the control. On the contrary, with EHDPP at 150 mg/kg/d and in the positive control, both ALT and AST activities increased significantly (*p* < 0.01).

### 3.2. Induction of Hepatic DNA Damage in Mice Orally Exposed to EHDPP

**Figure 3:**
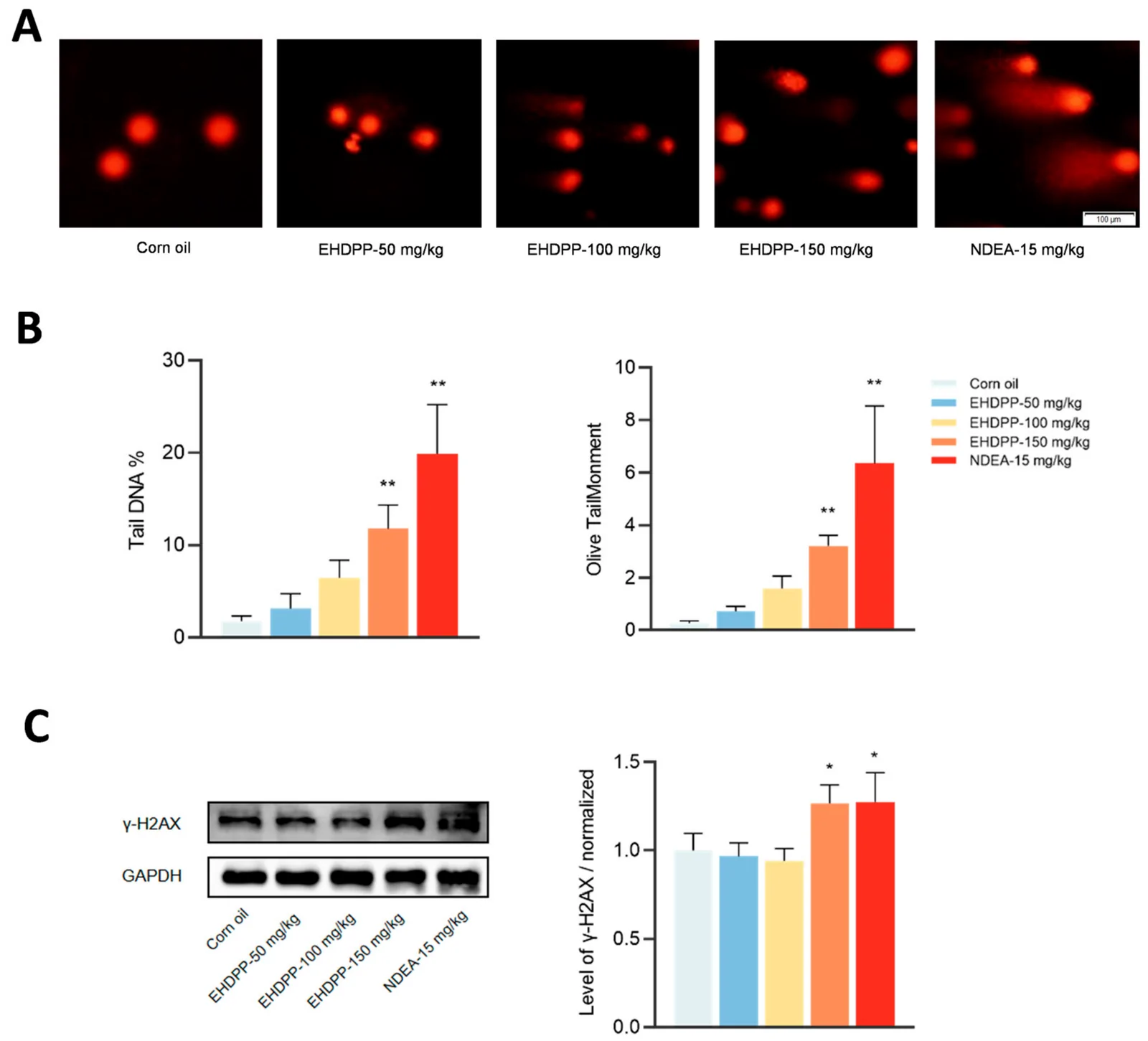
Induction of hepatic DNA damage by EHDPP as indicated by a comet assay (**A**,**B**) and hepatic γ-H2AX elevation (**C**). See the legend of Figure 2 for the chemical treatment regimen. On day 8 (within 24 h after termination of EHDPP exposure) fresh hepatocytes were isolated from each mouse, which were subjected to comet assay and Western blot assay of hepatic γ-H2AX. Data are means ± S.D. (n = 5); * *p* < 0.05, ** *p* < 0.01, by one-way ANOVA, as compared with the solvent control.

The DNA-damaging effect of test compounds were determined by using comet assay with hepatocytes isolated from each mouse and analyzing hepatic γ-H2AX by Western blot assay. As shown in Figure 3 (panels A & B), orally administered EHDPP at the highest dose (150 mg/kg/d) increased both the Tail DNA % and the Olive Tail Moment, as analogous to the effect in the positive control. This indicated a DNA-breaking effect of EHDPP. Meanwhile, for the 50 and 100 mg/kg/d groups both parameters from the comet assay also showed an increasing tendency, but with no statistical significance as compared with the control (*p* > 0.05). Likewise, the hepatic γ-H2AX levels in the group with 150 mg/kg/d of EHDPP were increased with statistical significance as compared with that in the control (similar to the response in the positive control), while no significant changes were observed in the groups with lower doses of EHDPP (see Figure 3C).

### 3.3. Results of Pig-a Gene Mutation Test in Mice Orally Exposed to EHDPP

**Figure 4:**
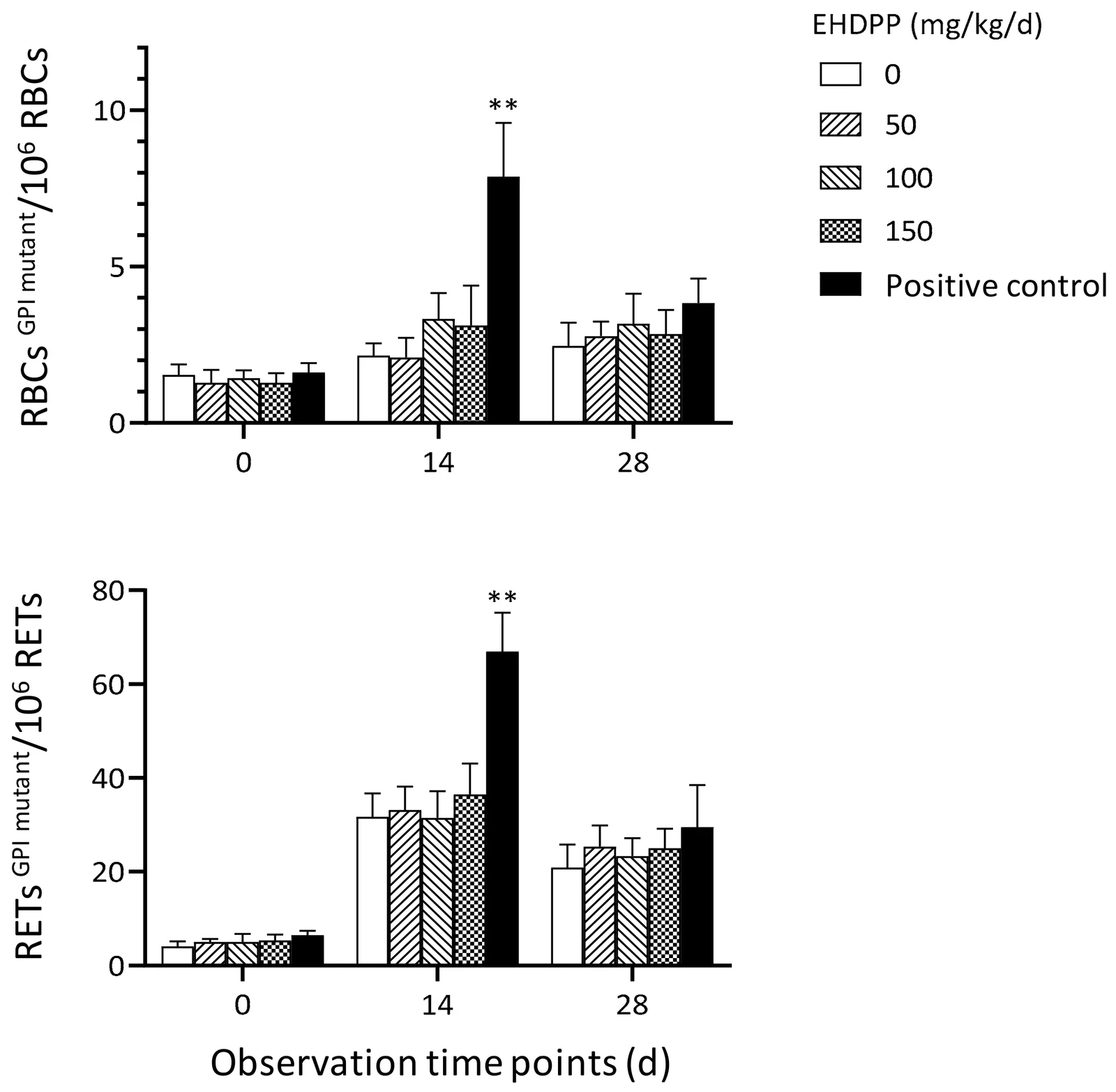
Results of Pig-a gene mutation test in circulative normal erythrocytes (**upper panel**) and reticulocytes (**lower panel**) in mice orally exposed to EHDPP on days 0, 14, and 28. See the legend of Figure 2 for chemical exposure regimen. The GPI-negative cells represent glycosylphosphatidylinositol (GPI) defective mutants. Data are means ± S.D. (n = 3); ** *p* < 0.01, by Student’s *t*-test, as compared with the control.

The levels of GPI-negative mutants were determined at three different time points, i.e., days 0, 14, and 28. As shown in Figure 4, the frequencies of mutants, in both the circulative erythrocytes (upper panel) and reticulocytes (lower panel), from mice in the positive control group at 14 d were significantly increased as compared with those in the (solvent) control (*p* < 0.01 in both cell types), which is consistent with existing reports. On the contrary, the frequencies of mutants at none of the time points in mice orally exposed to EHDPP at 50, 100, and 150 mg/kg/d for 7 d were significantly different from the relevant controls.

### 3.4. Induction of Micronucleus in the Bone Marrow of Mice Orally Exposed to EHDPP

As indicated in Table 1, in mice orally exposed to EHDPP at 150 mg/kg/d for 7 d, as similar to that in the positive control, the frequencies of micronucleus formation in bone marrow polychromatic erythrocytes were increased with statistical significance; while in the groups administered with lower doses of EHDPP (50 and 100 mg/kg/d for 7 d) no significant changes in the frequencies of micronucleus formation were observed, though the absolute values did gradually increase along with the elevation of doses.

### 3.5. Effect of EHDPP on Hepatic Protein Expression of AhR, PXR, Cyp1a1, Cyp1a2, Cyp2e1, and Cyp3a11

**Figure 5:**
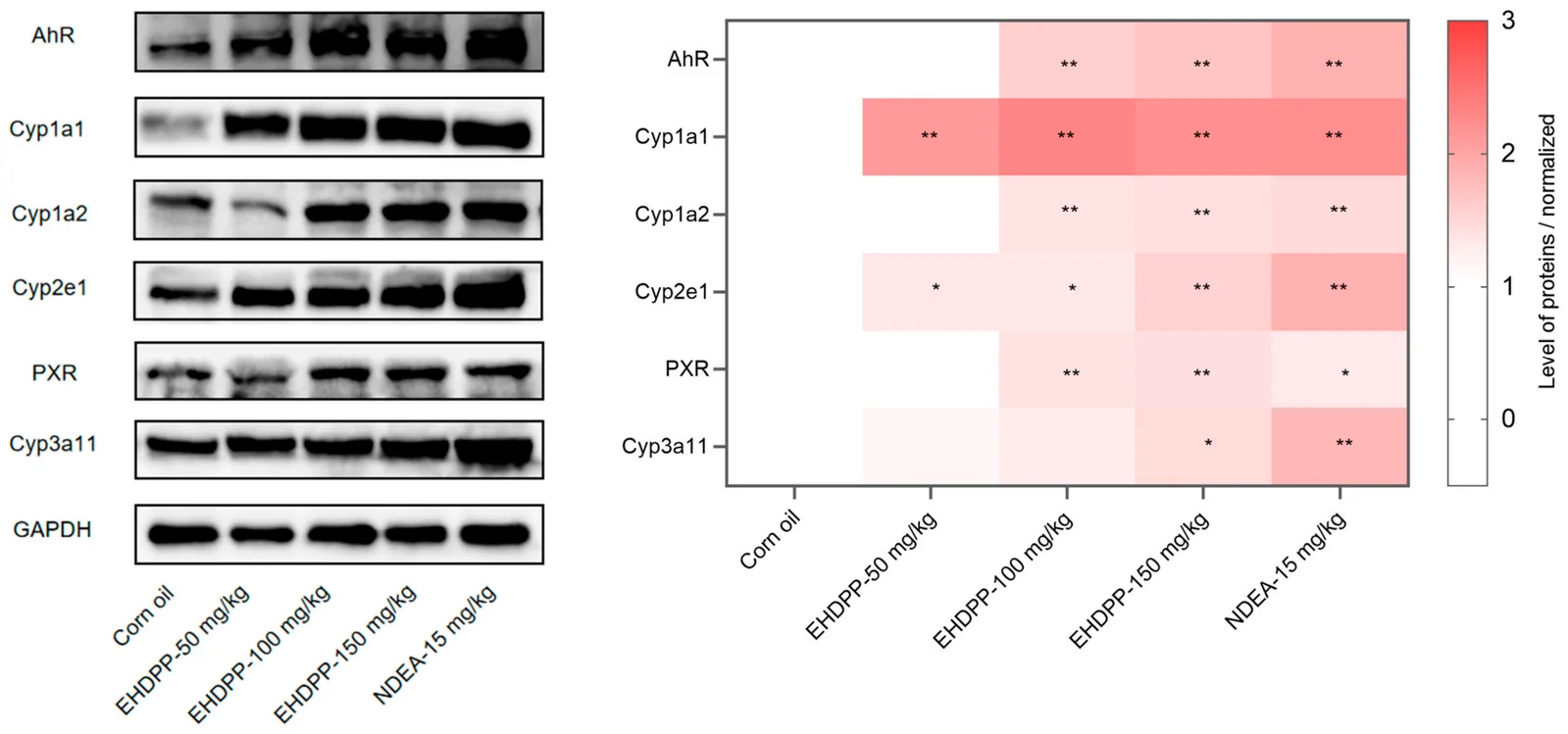
Levels of hepatic AhR, PXR, Cyp1a1, Cyp1a2, Cyp2e1, and Cyp3a11 proteins (by Western blot assay) in mice orally exposed to EHDPP for 7 d. See the legend of Figure 2 for the chemical exposure regimen. Values are means ± S.D. (n = 5); compared with the control (corn oil), * *p* < 0.05, ** *p* < 0.01, by one-way ANOVA.

As shown in Figure 5, in regard to EHDPP-induced changes in several hepatic proteins, (1) all six proteins, i.e., AhR, PXR, Cyp1a1, Cyp1a2, Cyp2e1, and Cyp3a11, increased in expression levels; (2) in regard to the relative potency and intensity of effect, the increase in Cyp1a1 protein expression was clearly most outstanding, followed by Cyp2e1, AhR, Cyp1a2, PXR and Cyp3a11 (in a potency/intensity decreasing order); (3) the paralleling positive control (NDEA at 15 mg/kg/d) also induced Cyp1a1 most efficiently, in the order of Cyp1a1 > AhR/Cyp2e1/Cyp3a11 > Cyp1a2/PXR.

### 3.6. Cytotoxicity and Micronucleus Formation by EHDPP in Hepa1-6 Cells and the Influence of AhR Modulators

**Figure 6:**
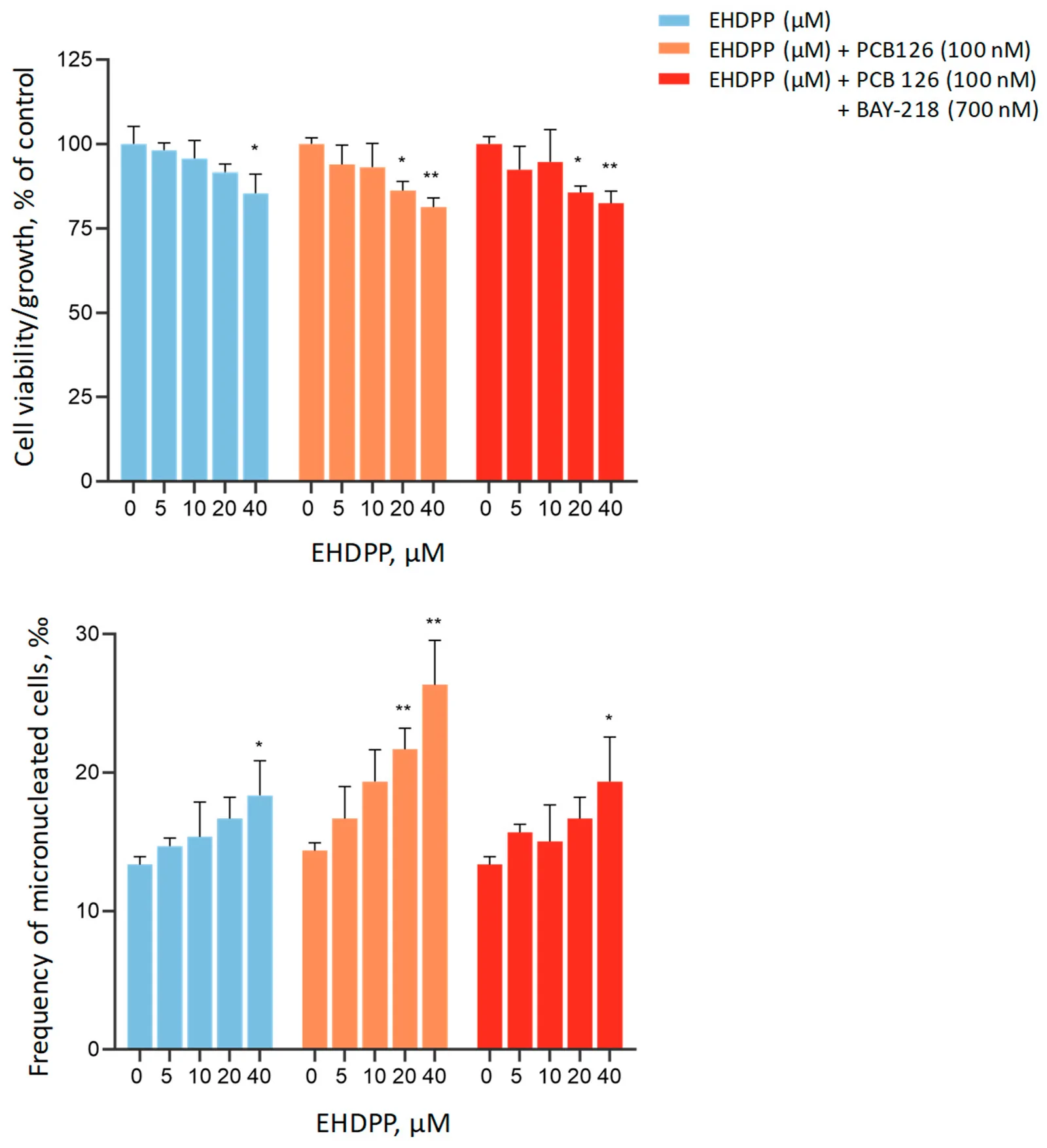
Cytotoxicity and micronucleus formation by EHDPP in Hepa1-6 cells and the impact of PCB 126 and BAY-218 as AhR modulators. Hepa1-6 cells were exposed to EHDPP at concentrations of 5, 10, 20, and 40 μM for 48 h, with 6 replicates in the CCK-8 assay and duplicate experiments in the micronucleus test. PCB 126 (0.1 μM) and BAY-218 (0.7 μM) were present in the cultures from 18 h ahead of EHDPP exposure to the end of test regimen (66 h in total). Data are means ± S.D. for the cell viability/growth values, while the micronucleus test results are expressed as means ± 1/2 ranges of variation. As compared with the control, * *p* < 0.05, ** *p* < 0.01 (see Section 2.12 for the statistical analytical methods).

As indicated in Figure 6 (upper panel), EHDPP alone did not change the cell viability/growth at concentrations from 5 to 20 μM, while at 40 μM a mild reduction in cell viability/growth occurred. In the presence of modulator PCB 126 (100 nM) and the combination of both PCB 126 and BAY-218 (700 nM), the threshold concentration of EHDPP for a reduction in cell viability/growth became a little lower, i.e., 20 μM, indicating mildly increased potency of EHDPP cytotoxicity. However, the cytotoxicity of EHDPP, either with or without modulators, stayed mild, with the relative cell viability/growth levels kept above 80%.

In the micronucleus test (Figure 6, lower panel), EHDPP alone did not influence the frequency of micronucleated cells, except for the highest concentration (40 μM) where it was increased with statistical significance. With PCB 126 (100 nM) as a modulator, EHDPP increased the frequency of micronucleated cells along with the elevation of its concentration, which met statistical significance at 20 and 40 μM, in a concentration-dependent manner. However, further addition of a second modulator, BAY-218 (700 nM) led to alleviated micronucleus formation, as indicated by the increase in the threshold concentration back to 40 μM (indicating a negative modulation on micronucleus formation).

## 4. Discussion

In this study, treatment of mice with EHDPP at 50~150 mg/kg/d doses for 7 d did not show obvious influence on the general condition, physical activities, body weight gain, or the liver organ coefficient, while a dose-dependent hepatotoxic effect was observed with EHDPP from 50 to 150 mg/kg/d; however, only at the highest dose (150 mg/kg/d) was elevation of serum aminotransferase activities present. It appears that EHDPP at 150 mg/kg/d may induce mild liver damage, and at lower doses the pathologic changes might be too limited (in distribution or intensity) to significantly increase the leakage of amino transferases into the blood. Also, at a dose of 150 mg/kg/d EHDPP, both hepatic DNA damage and bone marrow micronucleus formation were induced. The Pig-a assay with EHDPP for either circulative erythrocytes or reticulocytes was negative; although the 7-day exposure and 14-day and 28-day check points of Pig-a mutations in rodents are in accordance with the guideline for the Mammalian Erythrocyte Pig-a Gene Mutation Assay set by the OECD [21], the validity of this negative result is challenged by the very small sample size, which needs to be clarified in future studies with larger (≥5 animals per group) sample sizes, particularly using sensitive methods such as the transgenic rodent mutation assay. Meanwhile, in this study only male mice were used, so the results cannot be generalized to female animals, as Cyp expression, nuclear receptor activity, and susceptibility to hepatotoxicity could be sex dependent. The hepato- and genetic toxicity dose of EHDPP of 150 mg/kg/d under the current subacute exposure model is 25-fold and 7.5-fold higher than the no adverse effect levels (NOAEL) in rats under oral exposure for 90 d (subchronic) (6 mg/kg/d) [28] and 28 d (repeated dose) (20 mg/kg/d) [29], respectively. Whether and to what extent chronic exposure may potentiate the hepato- and genetic toxicity of EHDPP in rodents need to be further explored.

In this study, the impact of EHDPP on the expression of several hepatic nuclear receptors and Cyp enzymes is associated with the most obvious induction of Cyp1a1, and secondly that of Cyp2e1. There has been no evidence suggesting an association of the expression of Cyp2e1 with AhR, however, AhR is an essential and transcriptional regulator of Cyp1a1 and a moderate regulator of Cyp1a2 in both the mouse [30,31] and humans [32,33]. Truly, in this study EHDPP induced the expression of Cyp1a2 less potently than that of Cyp1a1. PXR was also induced by EHDPP in our study, with lower intensity than AhR. Cyp3a11 (and its human homologue CYP3A4) is downstream of PXR on a transcriptional regulation axis [34,35], and indeed hepatic Cyp3a11 in mice was also induced by EHDPP, though only at its highest dose (150 mg/kg/d). In both human-hepatocyte-derived cell models and in the liver of intact adult male C57BL/6J mice, PXRs were observed to inhibit the AhR/Cyp1a1 pathway and the relevant genotoxic effect of benzo(a)pyrene [36]. Interestingly, we have previously observed genotoxicity of EHDPP in mammalian cells, which depended on metabolic activation primarily by CYP3A4 and CYP2E1; the effect was almost blocked by ketoconazole [20], an inhibitor of PXR [37] and CYP3A4 [38]. Paradoxically, in this study induction of hepatic Cyp1a1 (and its upstream regulator AhR) by EHDPP apparently surpassed that of Cyp3a11 (and PXR). This prompted us to hypothesize that there might be a major difference between the mouse and humans in regard to the ability of AhR or PXR to be responsive to EHDPP, i.e., possibly the AhR/Cyp1a1 pathway is involved in activating EHDPP for its genotoxic effects in mice.

To test the above hypothesis, we designed an in vitro micronucleus test using the Hepa1-6 cell line. Our results indicated that PCB 126 as an AhR activator (at its non-genotoxic concentration, 100 nM) (supposedly enhancing the transcription of Cyp1a1) [39] enhanced the genotoxicity of EHDPP, while this effect was blocked by further addition of BAY-218 (700 nM, an AhR antagonist, which was expected to reduce Cyp1a1 expression). The results imply that Cyp1a1 might be involved in converting EHDPP into genotoxic metabolite(s) and a major difference could exist between the mouse and humans in the isoform of the P450 enzyme involved primarily in metabolically activating EHDPP. Nevertheless, this suggestion needs to be further clarified by continued, particularly in vivo, studies with selective Cyp modulators or specific gene knockout animals, as well as chemical identification of the involved reactive metabolites. In an in vitro metabolic system with human liver microsomes, a series of phase I metabolites of EHDPP have been identified, which include mono- and dihydroxylated metabolites (either on the alkyl chain or the phenyl group), keto metabolites, and mixed keto and hydroxylated metabolites, in addition to DPEP and EHMPP; meanwhile, phase II metabolism of many phase I metabolites and the formation of glucuronidated and sulfated metabolites were also observed [40]. While some of the hydroxylated and keto metabolites could be further oxidized by CYP enzymes to form more bioreactive (while more short-lived and more difficult to detect) metabolites, which may be critical for the genotoxicity of EHDPP, the phase II products from glucuronosyl and sulfo-conjugation may represent a detoxifying mechanism. Regretfully, our current technical capabilities do not permit identification of those metabolites, which has to be performed in the future.

It has been reported that EHDPP induced a series of toxicities in cultured mouse oocytes, including disrupted oocyte maturation and cell cycle progression, cytoskeletal damage, and accumulation of reactive oxygen species (ROS) [41]; however, whether these effects could be attributed to the proto-compound, the metabolites, or the combination of both remains unclear. In 6-week-old male CD-1 mice orally exposed to EHDPP at doses of 1, 10, and 100 mg/kg/d for 6 weeks, changes such as inhibited cell proliferation, enhanced apoptosis and oxidative stress in the testes and spermatocytes were observed, and ROS production appeared to cause DNA damage and mitochondrial dysfunction in the spermatocytes [42]; again, the relationship between metabolism of EHDPP and its effects was not studied. To the best of our knowledge, the present study is the first evidence for EHDPP-induced DNA and chromosome damage in intact mammalian animals and the potential relevance to metabolic activation. Meanwhile, besides the relevance of metabolic activation of EHDPP to its hepato- and genetic toxicity, the accumulation of ROS and inflammatory reactions by EHDPP, as already being observed in various in vitro and in vivo models [43,44,45], may also cause the observed toxic effects. This is consistent with the observation that BAY-218 as an antagonist of Cyp1a1’s upstream regulator (AhR) did not completely block PCB 126-potentiated micronucleus formation by EHDPP.

In regard to the hepatotoxicity of EHDPP, in a human hepatocyte (L-02) cell culture EHDPP disturbed the energy homeostasis and cell cycle and caused endoplasmic reticulum stress, apoptosis, and inflammatory response [12]. In 7-day-old male chickens, decreased liver coefficient, hepatocyte damage (on the plasma membranes and mitochondria) and hepatic inflammation were observed 14~28 d after a single oral dose of EHDPP (800~3200 mg/kg), which were partially attributed to EHDPP-induced oxidative stress [46]. The present study provides further evidence for the hepatotoxicity of EHDPP, particularly in a mammalian animal model, at a potency close to its genotoxicity.

The estimated human exposure of EHDPP is generally far lower than the doses applied in this study. For example, in an investigation of the OPFRs in Chinese foodstuffs and estimation of dietary intake in a population in Jiangsu Province, China, EHDPP was demonstrated to be the most abundant OPFR with a mean concentration of 1.12 ng/g wet weight in various food categories, and based on the general Chinese dietary habits and relevant consumption data the estimated dietary intake (EDI) of EHDPP was 8.4 ng/kg/d (the 95th percentile being 18.9 ng/kg/d), the reference dose (RfD) was 600 ng/kg/d, and the hazard quotient was 0.014 (95th percentile being 0.032) [47]. In a study performed in Portugal, estimated daily intakes of EHDPP from dust ingestion averaged 1.5 ng/kg/d for adults while 18 ng/kg/d for children [48]. Taken together, the levels of daily exposure of humans to EHDPP should be several orders lower than those in this study. While this may limit the possibility for EHDPP alone to induce hepatotoxicity or genotoxicity in humans under real life exposures, in our study the subacute exposure model may not represent the real life exposure, which is characterized by long-term and low-dose exposure, potentially permitting more potent induction of toxic effects. Meanwhile, considering that there are numerous OPFRs besides of EHDPP, as well as other kinds of organic pollutants (such as PBDEs, PCBs, and polycyclic aromatic hydrocarbons (PAHs)) which the human body may be exposed to simultaneously [49,50], there might be complex combined and chronic effects in which the potency of EHDPP may be further enhanced.

In conclusion, this study provides evidence for the hepatoxicity and DNA- and chromosome-damaging effects of EHDPP in mammalians; meanwhile, EHDPP may predominantly induce hepatic Cyp1a1, Cyp2e1 and AhR, and the AhR/Cyp1a1 pathway might be involved in activating EHDPP for genotoxicity. The toxic effects of EHDPP in the real world, characterized by exposure levels several orders lower, may be clarified by further investigations.

## Figures and Tables

**Table 1:** Induction of micronucleus formation in the bone marrow polychromatic erythrocytes (PCEs) in male mice exposed to EHDPP for 7 d.

Group	Dose (mg/kg/d)	Frequency of Micronucleated PCEs (‰)
EHDPP	0 (Corn oil)	2.63 ± 0.63
	50	3.25 ± 0.96
	100	3.40 ± 1.55
	150	4.40 ± 1.03 *
Positive control (NDEA)	15	7.80 ± 1.18 **

In each group, 5 mice were used, where 2000 randomly encountered PCEs were evaluated microscopically at a magnification of 1000 (under oil lens) for the presence of micronucleus. Data are means ± S.D., * *p* < 0.05, ** *p* < 0.01, as compared with the control using one-way ANOVA.

## Data Availability

The original contributions presented in this study are included in the article. Further inquiries can be directed to the corresponding authors.
